# Influence of Date Ripeness on Glycemic Index, Glycemic Load, and Glycemic Response in Various Saudi Arabian Date Varieties

**DOI:** 10.7759/cureus.48433

**Published:** 2023-11-07

**Authors:** Abdullah M Alzahrani, Khalid Alghamdi, Abdulaziz Bagasi, Osama A Alrashed, Abdulrhman F Alqifari, Hassan Barakat, Metab Algeffari

**Affiliations:** 1 Department of Family Medicine, King Saud Bin Abdulaziz University for Health Sciences, Jeddah, SAU; 2 Department of Family Medicine, King Abdulaziz Medical City, Jeddah, SAU; 3 Department of Family Medicine, King Fahad Armed Forces Hospital, Jeddah, SAU; 4 Department of Family Medicine, Ministry of National Guard Health Affairs, King Abdulaziz Medical City, Jeddah, SAU; 5 Department of Family and Community Medicine, King Fahd Specialist Hospital, Buraydah, SAU; 6 Department of Food Science and Human Nutrition, College of Agriculture and Veterinary Medicine, Qassim University, Buraydah, SAU; 7 Department of Family and Community Medicine, College of Medicine, Qassim University, Buraydah, SAU

**Keywords:** date, date maturity stages, glycemic indices, healthy diet, ripeness

## Abstract

Background

Dates have a special position in Middle Eastern countries, especially Saudi Arabia, and are essential to Arabic and Muslim diets. They are eaten in different forms according to their stage of maturation. In this study, we aimed to estimate the glycemic index (GI), glycemic load (GL), and glycemic response of different stages of date fruit maturation.

Materials and methods

This prospective clinical trial was conducted at King Abdul-Aziz Medical City, Jeddah, Saudi Arabia. Thirteen healthy participants, seven males and six females, received 50 g of glucose as reference food and 50 g of equivalent carbohydrates from three samples of Khalas dates and three samples of Barhi dates at different maturation stages (Khalal, Rutab, and Tamer). The GI, GL, and glycemic response for each type was calculated.

Results

The calculated means±SD of GI of the different stages of date maturation were 60.57±25.93, which raged from 53±16.49 to 71.06±32.97. The Khalal stage had the highest GI value, while the Tamer stage had a low GI value of 69.14 and 53.09, respectively. The GL ranged from 7.81 to 18.81. The Rutab stage had the highest GL, whereas the Khalal stage had the lowest GL values of 17.66±6.94 and 9.64±4.72, respectively. There was no significant difference in GI between different date maturation stages (p = 0.48). However, the GL presented a significant difference (p = 0.001) between different maturation stages.

Conclusion

The present study demonstrated that the stage of date maturation can affect the GI, GL, and glycemic response results. Therefore, healthcare providers and dietitians should address the lower GI and GL stages of date maturation in choosing a suitable carbohydrate source for healthy and diabetic individuals.

## Introduction

The date palm (Phoenix dactylifera L.) is thought to have originated in Mesopotamia, an area that roughly corresponds to most of modern-day Iraq, Kuwait, parts of Saudi Arabia, the eastern regions of Syria, and southeastern Turkey [1]. The date palm has a special position in Middle East countries, especially Saudi Arabia. The world's date production has remarkably increased from 3.4 million tonnes produced in 1990 to 9.6 in 2021, representing 181% growth. The leading producer country is Egypt, followed by Saudi Arabia, with more than 1.7 and 1.5 million tonnes produced in 2021, respectively, accounting for 18% and 16% of the world's total production. This represents a significant increase since 1990, with production rising by 222% in Egypt and 197% in Saudi Arabia. The higher production of Egypt is mainly attributed to significantly higher tree density per hectare than that of Saudi Arabia. The next leading producers ranking in the top 10 countries in producing dates after the first two are Iran, Algeria, Iraq, Pakistan, Sudan, Oman, United Arab Emirates, and Tunisia, sharing 55% of the total world's production. Other countries share 5% of the world's date production, with each producing 1-2%. Libya, Morocco, Kuwait, Türkiye, and Yemen produced tonnes ranging from nearly 60,000 to more than 179,000 in 2021. Cultivation of dates has also moved across the world with the development of civilization into other regions such as European, American, and East Asian countries; China shares 2% of the total world's production, with more than 159,000 tonnes produced in 2021, while 1% goes for the United States with more than 53,000 tonnes produced in 2021. Other European and American countries such as Albania, Mexico, and Peru share less than 0.5% of the world's production, with roughly 14,000, 19,000, and 300 tonnes produced in 2021 [2,3]. The growth and development of date fruits involve a series of changes, primarily in their color and chemical composition. These transformations take place through five distinct stages known as Hababouk, Kimri, Khalal (Balah), Rutab, and Tamar. The terms of these stages are in Arabic words with no equivalent words in English, but they are widely used internationally [1]. The first stage is the "Hababouk stage," which starts soon after fertilization and takes four to five weeks to finish, and it is considered immature date fruit. The second stage is the "Kimri stage" or "Green stage," which is hard, green in color, and unsuitable for eating. It is the longest stage of date development and lasts nine to fourteen weeks, depending on the date palm varieties. The third stage is the "Khalal stage" or "color stage or Biser," the date fruit is mature, and the color changes from green to yellow or red depending on the date type. Finishing this stage takes three to five weeks. The fourth stage is known as the "Rutab stage," referred to as the soft ripe stage. During this phase, the dates turn a brown color, indicating increased moisture content, and become softer. This stage typically lasts two to four weeks. The final stage is the "Tamar stage," during which the dates acquire a darker coloration. At the Tamar stage, the date fruit reaches its maximum level of total solids and retains less than 25% moisture, in contrast to the Rutab stage, which has a higher water content of approximately 30-45%. The lower moisture content at the Tamar stage makes it ideal for storage as it reduces the risk of fungal spoilage post-harvest. In contrast, dates in earlier stages, like Khalal and Rutab, are more perishable due to their higher moisture levels. Dates are harvested and sold at three different stages of maturity, Khalal, Rutab, and Tamar, depending on the variety, the prevailing climatic conditions, and market demands. Dates are an essential component of Arabic and Muslim diets. Dates serve as a good source of many nutritious and healthy elements. Dates are a rich source of energy due to their high sugar content, primarily consisting of fructose and glucose, while being low in fat and protein. They also contain a variety of minerals, with selenium, copper, potassium, and magnesium being predominant. Additionally, dates are a good source of vitamins, including significant amounts of vitamins C and B [4-6]. Dietary recommendations focus on the quantity rather than the quality of the carbohydrate, although the source and nature of carbohydrates can profoundly affect postprandial glycemia. However, even when foods contain the same amount of carbohydrates, they can differ in the extent to which they raise blood glucose levels. This differential effect is measured by the glycemic index (GI) [7-8]. The GI can be defined as a measure of the relative impact of carbohydrate-containing foods on blood glucose. A specific food's GI can be determined by evaluating the incremental rises of blood glucose after eating a food that contains 50 g of available carbohydrates and then comparing it with the same amount of carbohydrates from a reference food. The general values applied to defining the GI of a particular food, using glucose as a reference, are low (GI: 55 or less), medium (GI: 56 to 69), and high (GI: 70 or more) [8,9]. The GI values have been used successfully to predict the glycemic response of a meal for individual foods [9]. The effect of low GI on type 2 diabetes mellitus and lipid profile is positive. In meta-analyses of trials comparing low and high GI diets, low GI diets significantly reduced glycated hemoglobin (A1C) and total and low-density lipoprotein (LDL) cholesterol [10-12]. However, the GI does not provide information on how to increase and prolong the glycemic response of a specific amount of food. The concept of glycemic load (GL) was developed to address both. It considers the GI and the portion size to provide information on a real-life impact on postprandial glycemia [13]. Several factors determine the GI of a food, including the type of carbohydrate, protein content, fat, pH, amount and type of fiber, and finally, the particle size of the food [12]. Carbohydrate-rich foods that break down quickly and are absorbed into the bloodstream are classified as high-GI foods, which leads to a rapid rise in blood glucose and an insulin response. Conversely, foods with a low GI have a slower and lower effect on postprandial blood glucose and insulin response levels, respectively [13]. Given that the GI does not provide information on the increase and duration of glycemia when consuming a certain amount of carbohydrate-rich food, a separate measure called the GL is used, which accounts for both and thereby provides a more accurate picture of a food's real-life impact on postprandial glycemia [13]. The term GL combines the GI of a food or diet with the amount of carbohydrates in a given amount of food, meal, or diet [14]. Most previous studies have indicated that the GI for various date varieties ranges from low to medium [14, 15]. However, these studies were limited because they only included dates in the Tamar stage, the final stage of maturation. At this stage, dates exhibit a lower moisture content and turn a darker brown or almost black color [14, 15]. In contrast, the Rutab stage exhibits a higher level of hydration [4]. A study conducted in the Emirates in 2003 suggested that this increased hydration in the Rutab stage might influence gastric emptying or intestinal absorption, thereby affecting the GI [16]. To our knowledge, no studies in Saudi Arabia have measured the GI of dates at various stages of maturation. The aim of our study is to estimate the GI, GL, and glycemic response at different maturation stages of dates to assist healthcare professionals with dietary recommendations.

## Materials and methods

**Figure 1:**
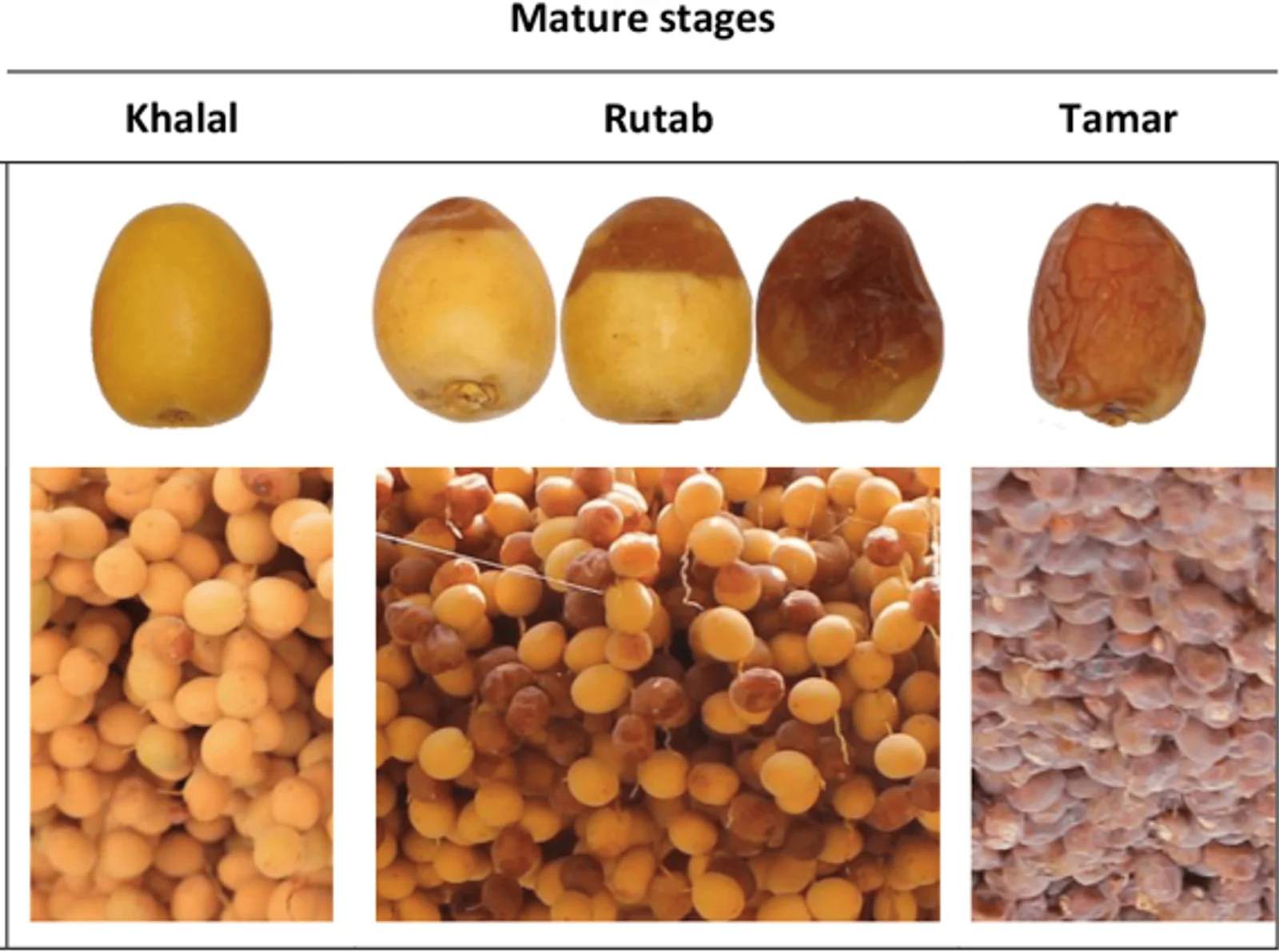
The three stages of Barhi dates' maturation: Khalal, Rutab, and Tamar. Adapted from reference 27.

The clinical trial was conducted at King Abdulaziz Medical City in Jeddah, Saudi Arabia. Healthy adults aged 18 to 60 were recruited through advertisements sent via student emails at King Saud Bin Abdulaziz University and to residents of Jeddah National Guard families. Exclusion criteria included volunteers with a history of diabetes, prediabetes, pregnancy, a BMI over 30, smoking or alcohol use, gastroenteritis in the last six months, gastroenterological disorders, surgeries of the alimentary tract, use of any medications, or a history of chronic diseases such as rheumatoid arthritis and bronchial asthma [14-16]. Institutional Review Board (IRB) approval was secured, and all participants were fully informed about the study's purpose and procedures. Their participation was voluntary, with the option to withdraw at any time. Each volunteer was afforded ample opportunity to inquire about their role in the trial before providing written consent. All volunteers underwent a health assessment to evaluate their health state, including measurement of their height, weight, BMI, presence of any diseases, previous surgeries, use of any medications, and smoking or alcohol use. Before measuring the GI, all volunteers underwent comprehensive health screenings, including normal fasting blood glucose, glycated hemoglobin (A1C), complete blood count (CBC), urea and electrolytes, liver function tests, lipid profiles, and oral glucose tolerance tests to exclude diabetes and other chronic conditions. Three stages of date maturation were tested: Khalal, representing the second stage; Rutab, representing the third stage; and Tamar, representing the fourth stage, from Khalas and Barhi date varieties (Figure 1). Dates were harvested in the same year from a local farm in Buraydah (Al Qasem region) and were free of any physical damage, insect injuries, or infestations. Khalas and Barhi varieties were chosen due to their availability at different stages of maturation and their popularity in Saudi Arabia, with Khalas being the most widely cultivated date palm variety [2, 3]. The date samples were processed by removing the kernels and then homogenizing the flesh to create a smooth paste. Using high-performance liquid chromatography, as per the modified Association of Official Agricultural Chemists (AOAC) method 982.14, levels of sucrose, glucose, fructose, and maltose in the date paste were determined [17,18]. The sugar profile was expressed in grams per 100 grams (g/100 g).

**Figure 2:**
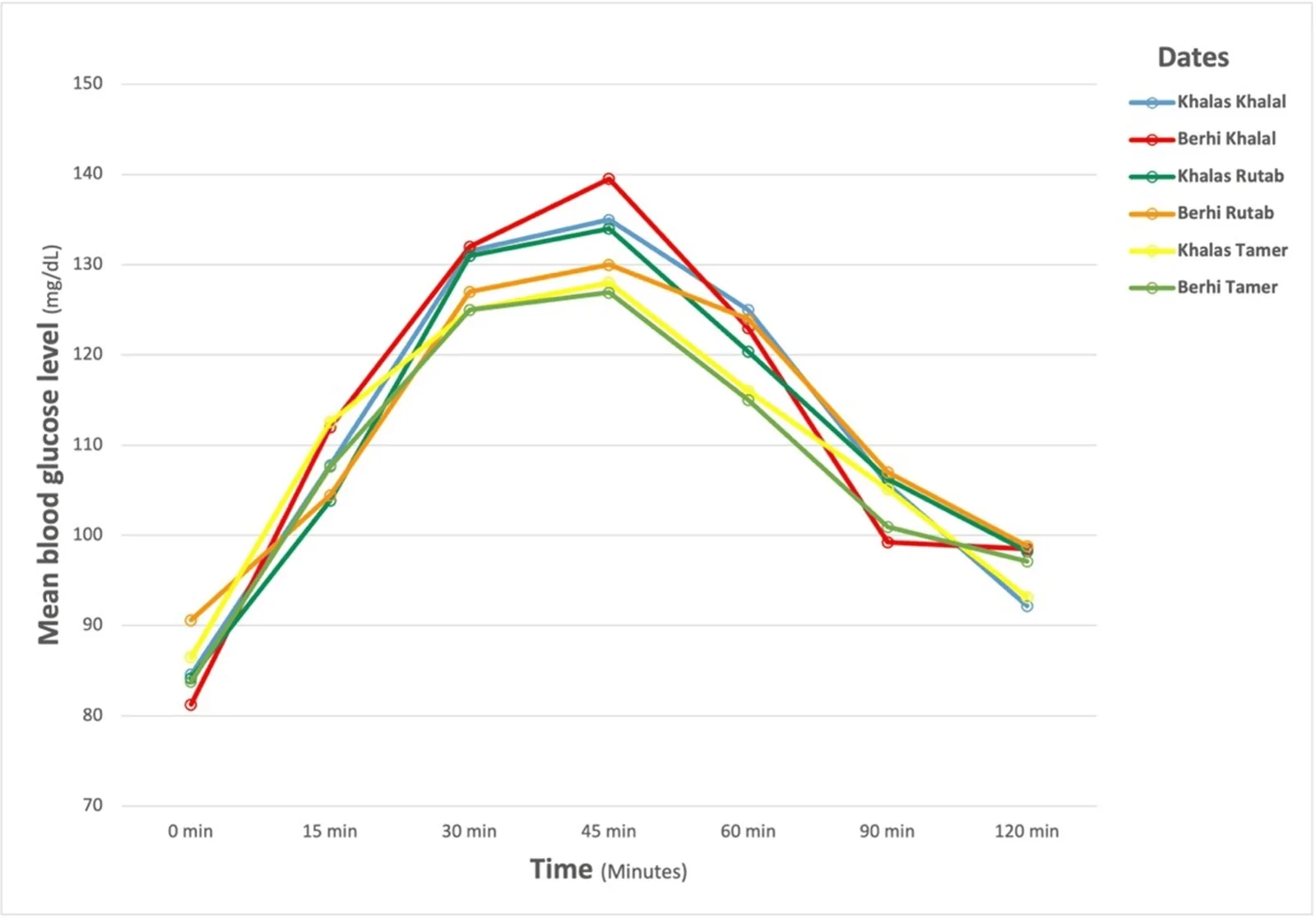
Mean blood glucose response of the subjects to the different date varieties.

The protocol of our study followed the methods described by Brouns F et al. and the Food and Agriculture Organization (FAO)/WHO, which indicate that the GI of food should be determined using tests repeated in at least six subjects; however, testing in ten subjects provides a higher degree of statistical power and precision [19, 20]. Thus, at least 10 subjects were used to analyze each food test. As a reference food, we used 50 g of glucose dissolved in 250 ml of water (Glutol-50, Saudi Medical Solution Co., Riyadh, Saudi Arabia). Each subject had a GI test after an overnight fast of 8-10 hours on two separate days, with at least one "washout" day separating each test from the other. The 50 g equivalent carbohydrates of the different types of tested dates were given blindly to the subjects in random order. The GI and GL were calculated according to Wolever TM et al. [21]. The dates were weighed using an analytical scale (H110 Sartor, Sartorius, Goettingen, Germany), and blood glucose was measured at 0 (fasting), 15, 30, 45, 60, 90, and 120 minutes. Blood glucose was measured using Freestyle Libre (Abbott Diabetes Care, Alameda, CA, USA), which has the US FDA approval for measuring blood glucose in adults (>18 years). According to the National Institute for Health and Care Excellence (NICE) guidelines, a study demonstrated device accuracy and acceptability ranging from 97% to 99% in comparison to venous blood sampling [21, 22]. Participants received training on how to use the Freestyle Libre and were instructed to remove the sensor prior to undergoing CT or MRI procedures. Additionally, they were cautioned against engaging in intense exercise and advised to abstain from taking ascorbic acid (vitamin C) while the sensor was in place (Figure 2) [23].

Data were analyzed using Microsoft Excel 16.6 and SPSS version 25 (IBM Corp., Armonk, NY, USA). GI and GL were measured for each date meal by calculating the area under the curve using an Excel spreadsheet provided by Prof. Thomas Wolever from the University of Toronto, Canada. GI values >2 SD above the mean were considered outliers and were excluded. The data are presented as means and SD. Since the values are continuous, the GI of the different date varieties was compared using ANOVA.

## Results

The number of subjects involved in the study was 13 (7 males and 6 females). The mean ± SD age of the subjects was 27.3±1.5 while the mean for the height and weight were 166.9 ± 7.1 and 71.1 ±16.2, respectively. The BMI of subjects had a mean±SD of 25.3 ± 4.5, while the mean for HBA1C was 4.9 ± 0.37, as shown in Table 1.

**Table 1:** Baseline demographics of the study subjects.

Mean (SD)	Variable
27.4 ± 1.56	Age (years)
166.9 ±7.1	Height (cm)
71.1 ±16.2	Weight (kg)
25.3 ±4.5	BMI (kg/m^2^)
4.9 ±0.46	Fasting blood glucose (mg/dL)
4.9 ±.37	HBA1C (%)

The quantity and type of sugars vary within cultivars and at stages of maturity. The reducing sugars ranged from 22.26% to 42.24%, 30.58% to 55.14%, and 38.31% to 64.91 at Khalal, Rutab and Tamer stages, respectively. In contrast, sucrose contents ranged from 6.58 to 15.44% in the Khalal stage and were undetectable in the Rutab and Tamer stages (Table 2).

**Table 2:** Sugar compositions of different types of dates.

Date	Glucose* (g/100 g)	Fructose (g/100 g)	Sucrose (g/100 g)
Khalas Khalal	7.75	7.67	6.58
Barhi Khalal	9.17	9.53	15.44
Khalas Rutab	28.91	30.76	Undetectable
Barhi Rutab	28.55	30.82	Undetectable
Khalas Tamar	26.74	22.64	Undetectable
Barhi Tamar	24.00	24.02	Undetectable

The calculated mean GI±SD of the date samples ranged from 60.57 ± 25.93 to 71.06 ± 32.97. Specifically, Khalas and Barhi Khalal varieties had the highest GIs, presenting as 71.06 ± 32.97 and 67.23 ± 34.08, respectively. Barhi and Khalas Tamar have the lowest GI, 53.2±17.11 and 53±16.49, respectively. The GL of the date samples ranged from 7.81 to 18.81. The Rutab stage had the highest GL as presented 18.81±8.08, 16.51±8.22), whereas the Khalal stage had the lowest GL (7.81±3.62 and 11.47±5.81). There was no significant difference in GI between different date maturation stages (p-value = 0.48). However, the GL difference between various stages was significant (p-value = 0.001). The calculated GI and GL results for all date stages are presented in Table 3.

**Table 3:** Mean glycemic index and glycemic load results.

Mean GL (SD)	Mean GI (SD)	Weight consumed in grams (g)	Number of subjects	Date type
7.81 ± 3.62	71.06 ± 32.97	227.27	10	Khalas Khalal
11.4 ± 5.81	67.23 ± 34.08	146.45	11	Barhi Khalal
18.81 ± 8.08	63.15 ± 27.11	83.79	12	Khalas Rutab
16.51 ± 8.22	55.8 ± 27.78	84.21	12	Barhi Rutab
13.04 ± 4.06	53.01 ± 16.49	101.25	12	Khalas Tamer
12.77 ± 4.11	53.19 ± 17.11	104.12	12	Barhi Tamer

## Discussion

Date palm has a special position in many Islamic regions, especially Saudi Arabia, and is considered an essential component in their diet. They are directly mentioned in the Muslim holy book, the Qur'an, and Hadith (by prophet Mohammed, peace be upon him). In the current study, we used one of the most widespread two types of dates (Khalas and Barhi) to measure their GI and GL in different stages of maturation (Khalas and Barhi Khalal representing the second stage, Khalas and Barhi Rutab representing the third stage, Khalas and Barhi Tamar describing fourth stage) [2, 3]. The mean GI of dates at the Tamar stage in this study was consistent with the previous studies, 53.09±0.13, range 53-53.19), which classified dates at the Tamar stage as a low GI item. In the study by AlGeffari MA et al., the mean GI of the 17 types of dates was 55.2 (range: 42.8-74.6). This range identifies dates as low to high GI. However, most dates had a low GI result [15]. In the study by Alkaabi JM et al., the GI range was 46-55 and 43-53 for healthy and diabetic participants, respectively, which classify dates as low GI food [16]. In another study that calculated the GI of three types of dates, Khalas, Barhi, and Bo ma'an, the GI range was 30.5-49.7 [24]. The previously mentioned studies had a limitation of including only one stage of date maturation (Tamar stage), while the current study involved different stages of date maturation: Khalal, Rutab, and Tamar stages. In the present study, the Khalal stage had the highest GI value, while the Tamar stages had a low GI value of 69.14 and 53.09, respectively. Additionally, considering the portion size by calculating the GL values could be a more practical measure [25]. The GL in this study significantly differed between the date stages (P<.001). The highest values were found in the Rutab stage, while the lowest was found in the Khalal stage (17.66 ± 6.94, 9.64 ± 4.72), respectively. Our study, in keeping with the previous studies, revealed that the level of hydration and fructose content of dates may affect GI results [15,17,26]. The high GI of dates at the Khalal stage can be attributed to their sugar composition, with the lowest fructose content, recorded at 7.67 g for Khalas Khalal and 9.53 g for Barhi Khalal, and the highest sucrose content, at 6.58 g for Khalas Khalal and 15.44 g for Barhi Khalal, respectively. According to the study by Brouns F et al. and other studies, the GI is influenced by the levels of fructose and sucrose [19]. Another factor that can explain the high GI of the Khalal stage is the hydration level. According to Miller CJ et al., the level of hydration at a date stage can affect intestinal absorption, gastric emptying, or insulin secretion [24]. The consumption of dates could be a concern in our country as it is one of the countries with the highest prevalence of diabetes. According to a previous meta-analysis of trials comparing low and high GI diets in diabetic patients, low GI diets significantly reduced glycated hemoglobin (A1C) by 0.4-0.5 percentage points [11,12]. Therefore, addressing the maturity level and its effect on the GI, GL, and glycemic response is essential during diabetic patients' dietary plan advice. The result of the current study could support that dates at the Tamar stage with a low GI index would be a suitable carbohydrate source for healthy and diabetic patients.

## Conclusions

The present study demonstrated that the stage of date maturation can affect the GI, GL, and glycemic response results. Therefore, healthcare providers and dietitians should address the stages of date maturation in choosing a suitable carbohydrate source for healthy and diabetic patients.
