# Correction: Influence of Date Ripeness on Glycemic Index, Glycemic Load, and Glycemic Response in Various Saudi Arabian Date Varieties

**DOI:** 10.7759/cureus.c498

**Published:** 2026-11-02

**Authors:** Abdullah M Alzahrani, Khalid Alghamdi, Abdulaziz Bagasi, Osama A Alrashed, Abdulrhman F Alqifari, Hassan Barakat, Metab Algeffari

**Affiliations:** 1 Department of Family Medicine, King Saud Bin Abdulaziz University for Health Sciences, Jeddah, SAU; 2 Department of Family Medicine, King Abdulaziz Medical City, Jeddah, SAU; 3 Department of Family Medicine, King Fahad Armed Forces Hospital, Jeddah, SAU; 4 Department of Family Medicine, Ministry of National Guard Health Affairs, King Abdulaziz Medical City, Jeddah, SAU; 5 Department of Family and Community Medicine, King Fahd Specialist Hospital, Buraydah, SAU; 6 Department of Food Science and Human Nutrition, College of Agriculture and Veterinary Medicine, Qassim University, Buraydah, SAU; 7 Department of Family and Community Medicine, College of Medicine, Qassim University, Buraydah, SAU

This article has been corrected to update Dr. Metab Algeffari’s affiliation to: Department of Family and Community Medicine, College of Medicine, Qassim University, Buraydah, Saudi Arabia.

